# Colonization of Beef Cattle by Shiga Toxin-Producing *Escherichia coli* during the First Year of Life: A Cohort Study

**DOI:** 10.1371/journal.pone.0148518

**Published:** 2016-02-05

**Authors:** Raies A. Mir, Thomas A. Weppelmann, Mauricio Elzo, Soohyoun Ahn, J. Danny Driver, KwangCheol Casey Jeong

**Affiliations:** 1 Department of Animal Sciences, Institute of Food and Agricultural Sciences, University of Florida, Gainesville, Florida, United States of America; 2 Emerging Pathogens Institute, University of Florida, Gainesville, Florida, United States of America; 3 Department of Environmental and Global Health, College of Public Health and Health Professions, University of Florida, Gainesville, FL, United States of America; 4 Food Science and Human Nutrition Department, Institute of Food and Agricultural Sciences, University of Florida, Gainesville, Florida, United States of America; Cornell University, UNITED STATES

## Abstract

Each year Shiga toxin-producing *Escherichia coli* (STEC) are responsible for 2.8 million acute illnesses around the world and > 250,000 cases in the US. Lowering the prevalence of this pathogen in animal reservoirs has the potential to reduce STEC outbreaks in humans by controlling its entrance into the food chain. However, factors that modulate the colonization and persistence of STEC in beef cattle remain largely unidentified. This study evaluated if animal physiological factors such as age, breed, sex, and weight gain influenced the shedding of STEC in beef cattle. A cohort of beef calves (n = 260) from a multi-breed beef calf population was sampled every three months after birth to measure prevalence and concentration of STEC during the first year of life. Metagenomic analysis was also used to understand the association between the STEC colonization and the composition of gut microflora. This study identified that beef calves were more likely to shed STEC during the first 6 months and that STEC shedding decreased as the animal matured. Animal breed group, sex of the calf, and average weight gain were not significantly associated with STEC colonization. The metagenomic analysis revealed for the first time that STEC colonization was correlated with a lower diversity of gut microflora, which increases as the cattle matured. Given these findings, intervention strategies that segregate younger animals, more likely to be colonized by STEC from older animals that are ready to be harvested, could be investigated as a method to reduce zoonotic transmission of STEC from cattle to humans.

## Introduction

Infections from Shiga toxin-producing *Escherichia coli* (STEC) are responsible for 2.8 million acute illnesses around the world every year [1] with more than 265,000 illnesses occurring in the United States [2]. Among STEC serotypes, *E*. *coli* O157:H7 is the most well-known and can cause a diverse set of pathologies including diarrhea, hemorrhagic colitis, thrombocytopenic purpura and hemolytic uremic syndrome [3]. Cattle are asymptomatic carriers of STEC and have been suggested to be the primary reservoirs of human illness, where zoonotic transmission occurs through several routes including contaminated food, fecal-oral contamination, and direct contact with animals [4, 5].

Since the majority of human infections by STEC likely originate from contaminated animal products, especially beef [6], it has been proposed that a reduction in fecal shedding of STEC by cattle may significantly reduce the incidence of human infections [7, 8]. Thus, the reduction of STEC at the pre-harvest level represents an opportunity to control the exposure to contaminated animal products and reduce transmission to humans [9]. However, many of the factors that affect the colonization dynamics of STEC in beef cattle remain unidentified, which makes the reduction of STEC at the pre-harvest level challenging.

In a recent study [10], animal factors such as age and parity were significantly associated with STEC shedding, with the highest prevalence in cattle two years of age and the lowest prevalence in heifers. However, since the sample population only included female cattle older than one year of age, the colonization dynamics of STEC during the first year of life were unable to be examined. Likewise, with the inclusion of only female cattle of a single breed, the previously reported findings that animal breed and sex could affect the prevalence of *E*. *coli* O157:H7 in cattle were also unable to be corroborated [11]. To further investigate age, sex, and breed as potential factors for the colonization of STEC in beef cattle during the first year of life, a prospective cohort of new-born beef calves was followed for one year.

Additionally, we hypothesized that the differences in STEC colonization between heifers and cattle could be the result of differences in the gut microflora. Though adhesion factors and virulence profiles that affect colonization of STEC among cattle have been studied in detail [12, 13], little attention has been given to the role of the diversity of bovine microflora. Metagenomic sequencing is a rapidly emerging as a tool for analysis of complex samples (fecal, rumen and soil) that defy conventional techniques and are able to identify microbes present at low concentrations [14]. Using metagenomic analysis, we hope to elucidate the role of microflora in STEC colonization by comparison of the composition of the microflora as the calves matured during the first year of life.

Our results indicated that animal age was associated with STEC shedding while breed, sex, or weight gain were not statistically significant factors in the likelihood of colonization by STEC during the first year of life. We found higher STEC prevalence and lower diversity of bovine microflora at early ages along with fundamental differences in the microflora composition between cattle that were colonized by STEC compared to those that were not.

## Materials and Methods

### Ethics Statement

Standard practices of animal care and use were applied to animals used in this project. The research protocols used in this study were approved by the University of Florida Institutional Animal Care and Use Committee (IACUC Protocol #: 201308027).

### Animal Management

The study was conducted over a period of two years on a cohort of beef calves from a multi-breed beef calf population derived from Brahman and Angus cattle. Calves were housed at the Beef Research Unit of University of Florida (Gainesville, Florida USA). This farm has a loose system of housing with average stocking density equal to 1.5 acres (0.6 Ha) per cow. Typically cows are bred from March to May and calving takes place from late December to February. In this study calves were followed from early age through their first year of life with samples collected four times, spaced evenly three months intervals. Calves were kept with their dams until weaning, which took place in August (7–9 months age) during the third sampling period. Calves were classified into six breed groups: breed group 1 (80 to 100% Angus and 0 to 20% Brahman), breed group 2 (60 to 79% Angus and 21 to 40% Brahman), breed group 3 or "Brangus" (62.5% Angus and 37.5% Brahman), breed group 4 (40 to 59% Angus and 41 to 60% Brahman), breed group 5 (20 to 39% Angus and 61 to 80% Brahman), breed group 6 (0 to 19% Angus and 81 to 100% Brahman). Body weights were recorded at birth and at the time each fecal sample was collected.

### Fecal Sample Collection

Fecal samples were collected in March (n = 259), June (n = 263), August (n = 261) and December (n = 193), representing the time at which calves were 1–3, 4–6, 7–9, and 10–12 months of age, respectively. Due to culling of calves for reasons unrelated to this study, only 193 calves were available for the December sample. The sampling scheme resulted in 188 animals that had four matched samples, which were used to assess colonization dynamics of STEC. Calves were categorized into three groups based on sex including bulls, steers (castrated males) and heifers. Fecal samples were collected from the recto-anal junction (RAJ) of calves using sterile cotton swabs, placed in a sterile 15 mL conical tube and all samples were transported on ice and processed the same day using the protocol described below.

### Identification of Shiga Toxin-Producing *Escherichia coli*

This study utilized a combination of culture-based and nucleic acid-based methods for the detection and enumeration of Shiga toxin-producing *Escherichia coli* (STEC) from the fecal samples. Fecal swabs were resuspended with 2 mL of Tryptic Soy Broth (TSB) broth and spread plated onto MacConkey agar (Becton Dickinson Company, MD, USA) after 10-fold serial dilutions (up to 10^6^). Plates were incubated at 37°C and examined after 24 hours for the enumeration of total *Enterobacteriaceae*. From the resulting plates, 20 pink lactose-fermenting colonies were selected randomly and purified on CHROMagar *E*. *coli* (CHROMagar, Paris, France). Ten blue *E*. *coli* colonies were subjected to colony PCR for the detection of *stx1* and *stx2* genes. A multiplex PCR was carried out using two sets of primers: KCP 11/12 for *stx1* (655 bp amplicon) and KCP13/14 (477 bp amplicon) for *stx2* gene. The primer sequence is as follows; KCP11 (5′-TGTCGCATAGTGGAACCTCA-3′), KCP12 (5′-TGCGCACTGAGAAGAAGAGA-3′), KCP13 (5′-CCATGACAACGGACAGCAGTT-3′) and KCP14 (5′-TGTCGCCAGTTATCTGACATTC-3′) [15]. The PCR reaction conditions were 95°C for 5 minutes; 30 cycles of 95°C for 30 seconds, 55°C for 30 seconds, and 72°C for 90 seconds; with a final extension at 72°C for 7 minutes. The amplified products were resolved by electrophoreses using a 1% agarose gel stained with ethidium bromide and visualized with a UV gel doc system (Bio-Rad, USA). For interpretation of PCR results, *E*. *coli* O157:H7 (EDL933) and DH5α were used as positive and negative controls, respectively. Strains were sub-grouped depending on the toxin genes. STEC positive was used to describe the strains carrying either or both *stx* genes and were further classified as *stx1* or *stx2*, while strains with both genes were designated as s*tx1/stx2* positive.

### Statistical Analyses

Simple logistic regression was used to determine if differences in herd prevalence were significantly different by cattle age as well as the association between the presence of STEC, *stx1*, s*tx2*, and *stx1/stx2* and the animal breed group (groups 1–6), and sex (bull, steer, or heifer), for the four sampling periods. Physical development was evaluated by the change in animal weight (kg) between sampling periods, which was used to determine animals that fell under the 25^th^, between the 25^th^ and 75^th^, and above the 75^th^ percentiles in weight gain. For the animals with four consecutive samples collected (n = 188), the results of the microbiological testing between two sampling dates was used to determine the proportion of cattle that were not previously colonized and did not become colonized (-/-), were not previously colonized and became colonized (-/+), those that remained colonized (+/+), and those animals that were previously colonized and are no longer colonized (+/-). Significant increases and decreases in prevalence between any two consecutive sampling periods were determined by using McNemar's test for matched pairs. All statistical analyses were conducted using STATA software package (STATA^®^ MP 11.2, StataCorp, College Station, Texas, USA) with a significance threshold of α < 0.05.

### Metagenomic DNA Extraction and Pyrosequencing

To understand the association of microflora with animal age and its effect on the STEC dynamics, metagenomic analyses of the fecal samples from male and female calves from all six breed groups was conducted using 454 pyrosequencing (Macrogen, South Korea). The samples were collected at four different times during the first year of life. The DNA was extracted from the fecal samples with PowerSoil DNA Isolation kit (MoBio Laboratories, Carlsbad, CA, USA) according to the manufacturer’s protocol. Library was prepared using PCR products according to the GS FLX plus library prep guide. Libraries were quantified using Picogreen assay (Victor 3). The emPCR, corresponding to clonal amplification of the purified library, was carried out using the GS-FLX plus emPCR Kit (454 Life Sciences). Briefly, libraries were immobilized onto DNA capture beads. The library-beads obtained were added to a mixture of amplification mix and oil and vigorously shaken on a Tissue Lyser II (Qiagen) to create "micro-reactors" containing both amplification mix and a single bead. Emulsion was dispensed into a 96-well plate and the PCR amplification program was run according to the manufacturer's recommendations. Twenty monogram aliquot of each sample DNA was used for a 50 ul PCR reaction. The 16S universal primers 27F (5′-GAGTTTGATCMTGGCTCAG-3′), 518R (5′-WTTACCGCGGCTGCTGG-3′) were used for amplifying of 16S rRNA genes. FastStart High Fidelity PCR System (Roche) was used for PCR under the following conditions: 94°C for 3 minutes followed by 35 cycles of 94°C for 15 seconds; 55°C for 45 seconds and 72°C for 1 minute; and a final elongation step at 72°C for 8 minutes. After the PCR reaction, the products were purified using AMPure beads (Beckman coulter) and sequenced using the following method by Macrogen Ltd. (Seoul, Korea). Following PCR amplification, the emulsion was chemically broken and the beads carrying the amplified DNA library were recovered and washed by filtration. Positive beads were purified using the biotinylated primer A (complementary to adaptor A), which binds to streptavidin-coated magnetic beads. The DNA library beads were then separated from the magnetic beads by melting the double-stranded amplification products, leaving a population of bead-bound single-stranded template DNA fragments. The sequencing primer was then annealed to the amplified single-stranded DNA. Lastly, beads carrying amplified single-stranded DNA were counted with a Particle Counter (Beckman Coulter). Sequencing was performed on a Genome Sequencer FLX plus (454 Life Sciences), and each sample was loaded in 1 region of a 70 mm-75 mm PicoTiter plate (454 Life Sciences) fitted with a 8-lane gasket.

### Selection of 16S rRNAs and Taxonomic Assignment

Using the basic local alignment search tool (BLASTN), all the sequence reads were compared to Silva rRNA database. Sequence reads which had sequence similarity with less than 0.01 E-value were admitted as partial 16S rRNA sequences. Non-16S rRNA sequence reads comprised less than 1% of all reads. Taxonomic assignment of the sequenced read was carried out using NCBI Taxonomy Databases. The five most similar sequences for each sequence read were found by their bit scores and E-value from the BLAST program. Needleman-Wunsch global alignment algorithm was used to find the optimum alignment of two sequences along their entire length. A pairwise global alignment was performed on selected candidate hits to identify the best aligned hit. The taxonomy of the sequence with the highest similarity was assigned to the sequence read. By the similarity, we assigned the taxonomy down to these taxonomical hierarchies; species with more than 97% similarity, genus 94%, family 90%, order 85%, class 80%, and phylum 75%.

### Operational Taxonomic Unit (OTU) Analysis for Community Richness

CD-HIT-OTU software was used for clustering. Mothur software was used for analyzing microbial communities and Shannon-Weaver diversity index and Simpson index were used for species diversity. Statistical analysis of the metagenomic data for the microflora diversity and richness within the calves for different ages was analyzed using SAS (Version 9.4) and R program [16] was used to generate heat maps for the various OTUs present in fecal samples. Mean proportions and 95% CIs were used to describe the changes in proportions of the 16S rDNA reads assigned to different OTUs present in the fecal samples. Mean proportions of the bacterial taxa (OTUs) within the study groups were analyzed using a generalized linear mixed model in SAS (version 9.4, SAS Institute Inc., Cary, NC, USA).

## Results

### High Proportion of Young Beef Calves (1–3 Months Old) Were Colonized with STEC

**Fig 1:**
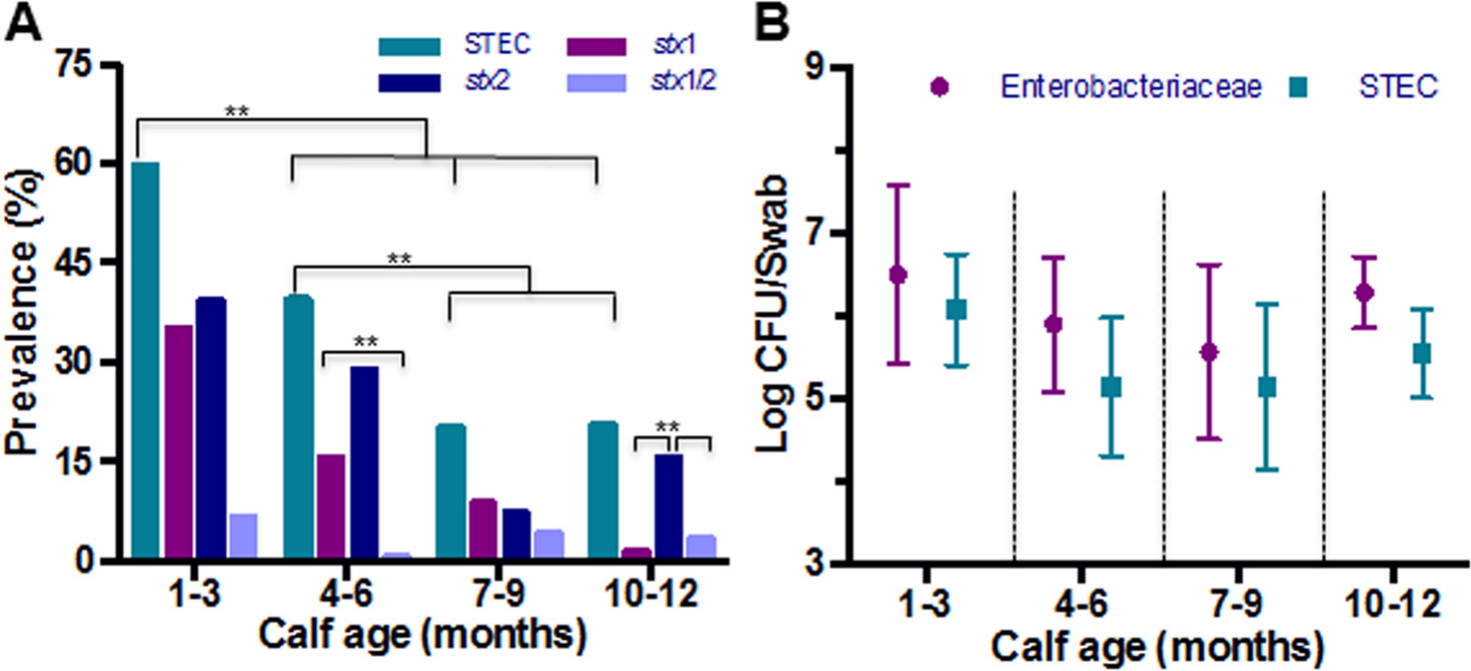
Prevalence and concentration of STEC in beef calves. (A) The prevalence of STEC, *stx1* genotype, *stx2* and *stx1/stx2* isolated from calves at four times during the first year of life are presented. For comparisons among age groups, *p*-values are included along with the level of statistical significance (*p*-value < 0.05 **). (B) The concentration (log CFU/swab) of STEC and *Enterobacteriaceae* isolated from calves at four times during the first year of life are presented. Statistical comparisons among age groups were examined using simple logistic regression to determine differences in prevalence (A), as well as simple linear regression to determine differences in bacterial concentrations recovered from recto-anal junction swabs (B).

The herd prevalence of STEC in young calves 1–3 months old was 60.3%, which was significantly higher compared to older calves (*P* < 0.001). As the calves matured, the prevalence decreased to 39.5% at 4–6 months age, 20.3% at 7–9 months age, and 20.7% in 12 months old calves (Fig 1A). The prevalence of *stx2* genotype was higher compared to *stx1* and *stx1/stx2* genotypes in all the four sampling times, except in 7–9 months old calves, where it was lower but not significantly different than *stx1* (7.4 vs 9.0%). The prevalence of *stx1/stx2* genotype was the lowest in all samplings except for the 12 month old calves. In contrast to the steady decline of animals colonized with the *stx1* genotype, the shedding of the *stx2* genotype began to increase at the age of 12 months, however only in female cattle (7.4 vs 14.9%).

The relationship between natural gut microflora on the STEC prevalence was shown by plotting the concentration of *Enterobacteriaceae* and STEC (log CFU) during the four sampling periods (Fig 1B). The concentration (log CFU) of *Enterobacteriaceae* and STEC shed by the calves was found to be normally distributed (Shapiro-Wilk normality test, P < 0.0001), allowing us to carry out further analysis on the concentration data. Calves shed the highest concentration of STEC in early age (1–3 month period) and as they matured, the colonization became less prevalent and the concentrations shed were lower. The concentration of *Enterobacteriaceae* shed remained the same among various age groups (Fig 1B). Reduced STEC prevalence with increased calf age was confirmed in the second year of sample collection wherein, the overall STEC prevalence at 9 and 12 months of age was 20.3% and 20.7%, respectively. Similarly, a higher proportion of the calves were shedding *stx2* genotype compared to the *stx1* or *stx1/stx2* genotypes at 9 and 12 months age in second year of sampling (data not shown).

### Association between Animal Factors and the STEC Prevalence

**Fig 2:**
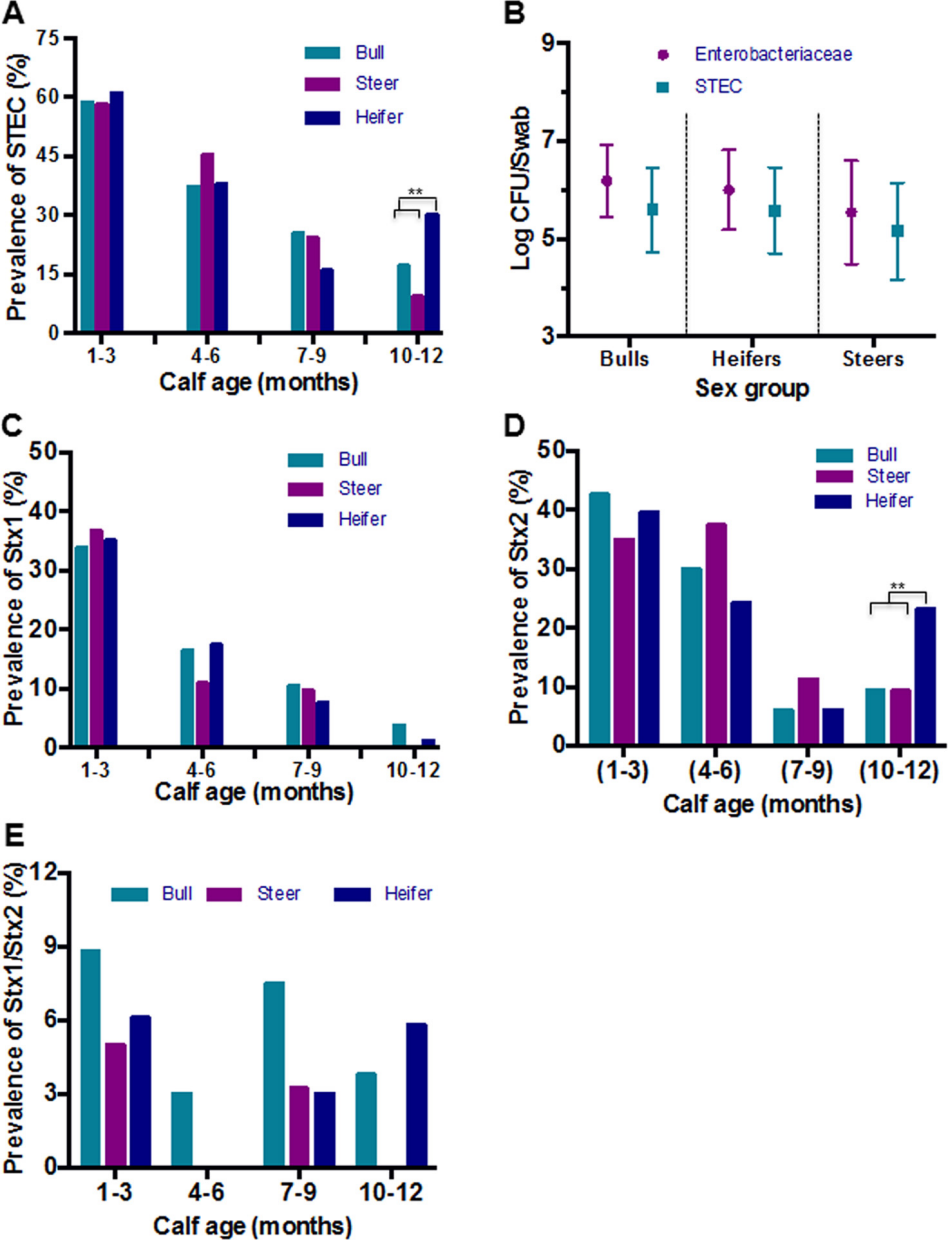
Prevalence of STEC among bulls, heifers and steers. **(A)** The prevalence of STEC in bull calves, steers and heifer calves during the first year of life is presented for any type of STEC and by individual genotype. Statistical comparisons between calves were examined using simple logistic regression to determine differences in prevalence. (B) Concentration of STEC and *Enterobacteriaceae* isolated from bull, steer, and heifer calves in four sampling points. (C) The prevalence of *stx1* genotype in bull calves, steers and heifer calves. (D) The prevalence of *stx2* genotype in bull calves, steers and heifer calves. (E) The prevalence of *stx1/stx2* genotype in bull calves, steers and heifer calves.

To understand if animal sex affects the prevalence of STEC, the animals were stratified by sex. Calves in all three groups (bull, heifer, steer) had higher prevalence of STEC shedding at 1–3 months of age and the prevalence of STEC generally decreased as they matured (Fig 2A). No significant association was found between calf sex and any STEC genotype. However, heifers had significantly higher rates of STEC shedding at 12 months age (OR = 2.11, *P* = 0.004) compared to their male counterparts, which actually increased from 6 months age from 15.9 to 30.2% (Fig 2A). There was no significant difference in the concentration of *Enterobacteriaceae* or STEC (of those animals that were positive) shed by the males and females, but the concentration of *Enterobacteriaceae* was higher than STEC (Fig 2B). The prevalence of *stx1* decreased with the age of calves irrespective of the sex (Fig 2C). The prevalence of *stx2* genotype was higher (43.1%) in young calves of 1–3 months of age and decreased with the age of the calf except in females, which actually increased at 10–12 months of age (Fig 2D). The prevalence of *stx1/stx2* genotype was very low in all age groups of the calves and the concentration was not associated with the sex of calf (Fig 2E).

**Fig 3:**
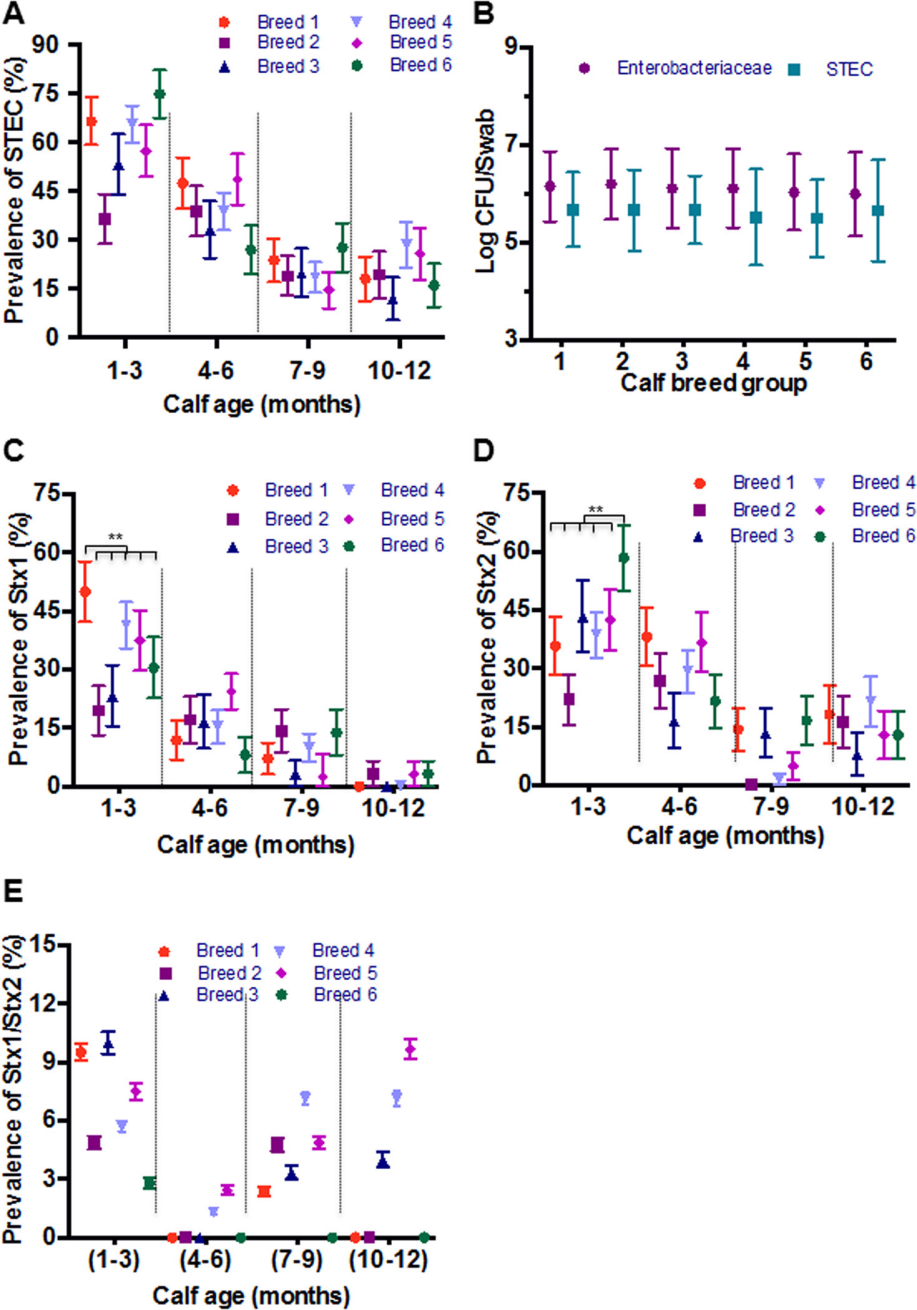
Prevalence of STEC among six breed groups. (A) The overall prevalence of STEC in six breed groups during the first year of life. (B) Concentration of STEC and *Enterobacteriaceae* isolated from six breed groups in four sampling points. (C) The prevalence of *stx1* genotype in six breed groups. (D) The prevalence of *stx2* genotype in six breed groups. (E) The prevalence of *stx1/stx2* genotype six breed groups. Statistical comparisons between calves were examined using simple logistic regression to determine differences in prevalence.

To understand if animal genetics affects the prevalence of STEC, the animals were stratified by breed groups. All breed groups showed a general trend of decreasing STEC prevalence from higher rates of colonization at 1–3 months of age to very low levels at one year age (Fig 3A). Overall breed was not significantly associated with STEC colonization, however there were some differences observed between breeds. No significant differences in the concentration of *Enterobacteriaceae* or STEC shed by the different breed groups were identified, even though the concentration of *Enterobacteriaceae* was higher than STEC (Fig 3B). There was a higher prevalence of *stx1* genotype in breed group 1 at 1–3 months of age while the prevalence remained low and was not significantly different among breed groups as the claves matured (Fig 3C). The prevalence of *stx2* genotype was higher in breed group 6 in 1–3 month old calves (*P* < 0.05), while the 4–6 month old calves of breed group 1 had a higher prevalence of *Stx2* (Fig 3D). The prevalence of *stx2* genotype decreased in all breed groups as the calves matured (Fig 3D). At each sampling point there was a very low prevalence of the *stx1/stx2* genotype that was not significantly different among the breed groups (Fig 3E).

**Fig 4:**
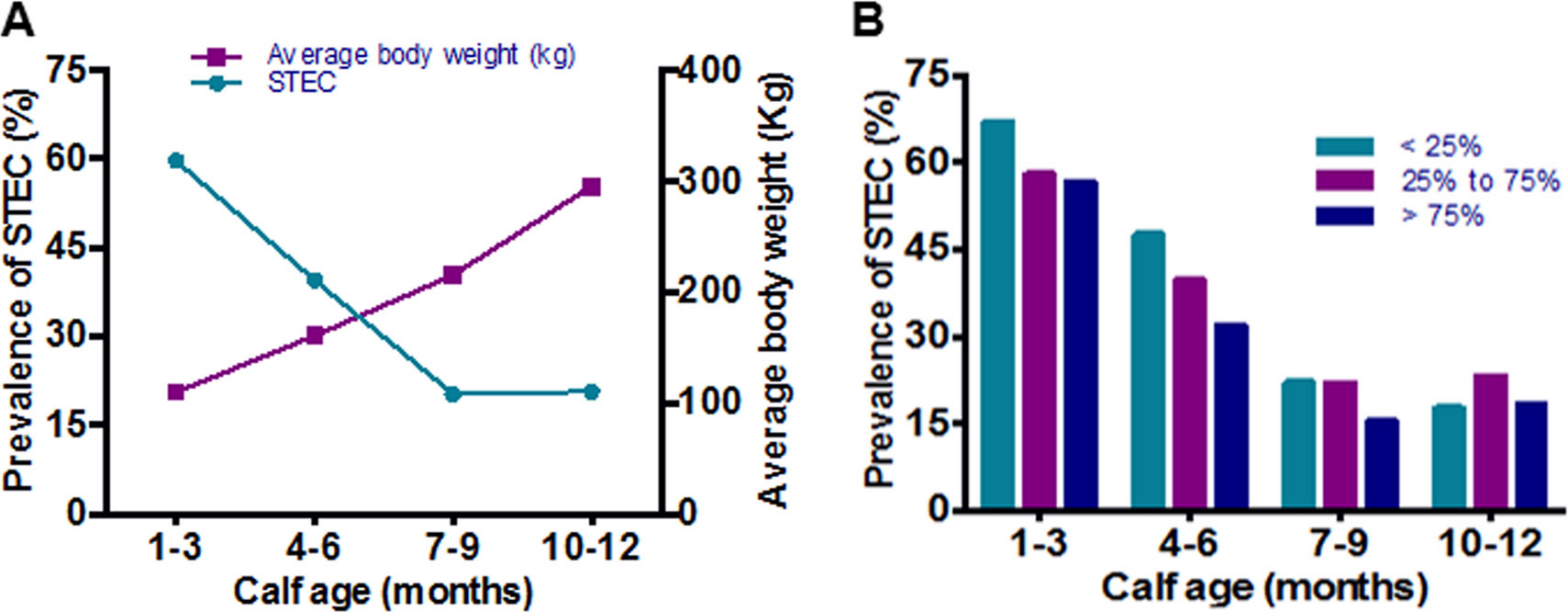
Prevalence of STEC and average body weight calves. (A) The prevalence of STEC in beef calves and average body weight (Kg) at different growing points in first year of life. (B) STEC prevalence with respect to gain in body weight. Physical development was evaluated by the change in animal weight between sampling periods, which was used to determine animals that fell under the 25th, between the 25th and 75th, and above the 75th percentiles in weight gain.

Cattle growth was also examined as a potential factor for STEC colonization. The average cattle weights show a steady linear growth pattern during the first year of life which inversely correlates with STEC prevalence in the herd (Fig 4A). After further stratification of the animal growth rates into < 25^th^, 25^th^ to 75^th^ and > 75^th^ percentiles, animals that gained more weight between sampling periods had no significant differences in the likelihood of becoming colonized with STEC, but the STEC prevalence was lower in animals that gained more weight (75^th^ percentile) (Fig 4B). There was also no significant association between breed group, sex of the calf, and average weight gain with the STEC shedding in the second year of sampling (data not shown).

### Colonization Dynamics of STEC in Calves

**Fig 5:**
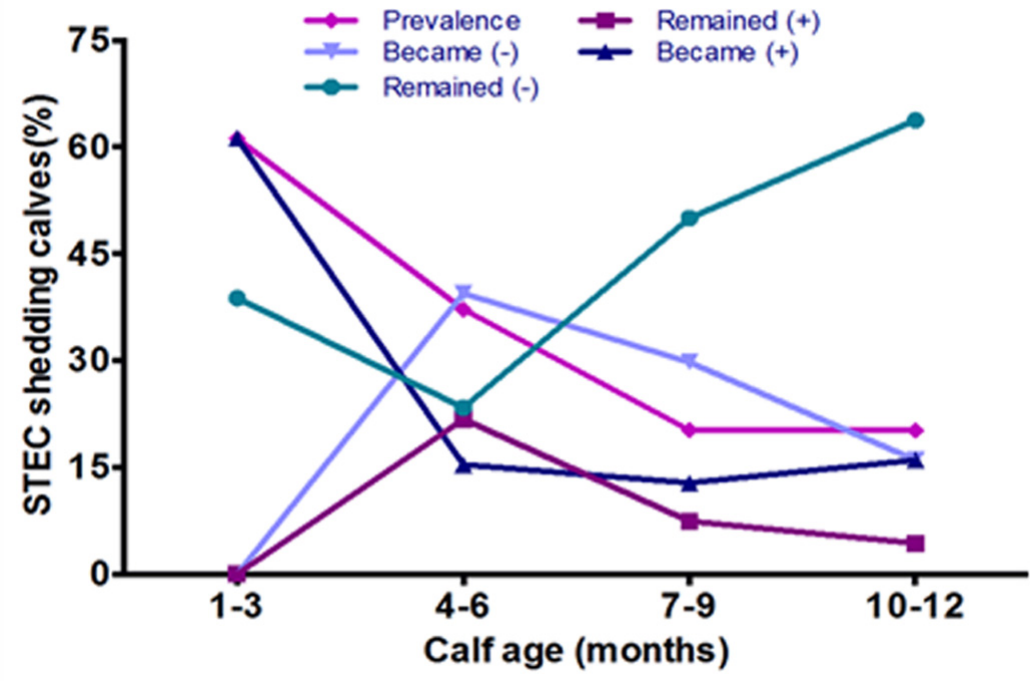
Dynamics of STEC in calves during the first year of life. For the animals with four consecutive samples collected (N = 188), the results of the microbiological testing between two sampling dates was used to determine the proportion of cattle that were not previously colonized and did not become colonized (-/-), were not previously colonized and became colonized (-/+), those that remained colonized (+/+), and those animals that were previously colonized and are no longer colonized (+/-). Significant increases and decreases in prevalence between any two consecutive sampling periods were determined by using McNemar's test for matched pairs.

Assuming that the calves were not colonized at birth, 61.2% of the animals had become colonized by the first sampling period at 1–3 months of age, followed by a net decrease of 23.9% driven primarily by a large proportion of previously colonized animals no longer shedding *stx1* (27.7%) and *stx2* (33.5%). Though 16.5% of cattle became colonized between 3–6 months of age, after the peak prevalence observed at 3 months age, the number of animals that were becoming colonized was much lower (Table 1). Furthermore, the number of animals which remained uncolonized after 3 months of age increased from 23.4% at 6 months age to 63.8% in one year old calves. Similarly, those animals colonized at 3 months age, 21.8% of them remained colonized at 6 months, followed by a marked decrease to only 7.4 and 4.3% remaining colonized at 9 and12 months of age, respectively. This high rate of colonization followed by a period of recovery from colonization leads to a relatively lower herd prevalence of STEC at the age of one year, where no net gain in animals becoming newly colonized was observed. These dynamics are shown graphically in (Fig 5), where after an initially high rate of colonization by STEC, a high proportion of the animals become no longer colonized and remain STEC negative. After the majority of cattle were no longer colonized by STEC, a much smaller proportion of the animals fluctuate between being colonized and not colonized, resulting in a stabilization of herd prevalence by the end of the first year of life.

**Table 1:** STEC prevalence and dynamics in beef calves^a^.

Prevalence of STEC by genotype						Colonization dynamics among samples^*b*^ (%)			
						*Gained*	*lost*	*remained*	*remained*
*Sample*	*Genotype*	*Prevalence*	*Diff*.	*P value*^*c*^	*OR*^*d*^	*(-)/(+)*	*(-)/(-)*	*(+)/(+)*	*(-)/(-)*
**Sample I**	**STEC**	61.2	61.2	-	-	61.2	-	-	38.8
**1 to 3**	***stx1***	32.4	32.4	-	-	32.4	-	-	67.6
**Months**	***stx2***	43.1	43.1	-	-	43.1	-	-	56.9
**March**	***stx1/2***	6.9	6.9	-	-	6.9	-	-	93.1
**Sample II**	**STEC**	37.2	-23.9	0.00**	0.39	15.4	39.4	21.8	23.4
**4 to 6**	***stx1***	14.4	-18.1	0.00**	0.35	9.6	27.7	4.8	58.0
**Months**	***stx2***	26.1	-17.0	0.001**	0.49	16.5	33.5	9.6	40.4
**June**	***stx1/2***	0.5	-6.4	0.001**	0.08	0.5	6.9	0.0	92.6
**Sample III**	**STEC**	20.2	-17.0	0.000**	0.43	12.8	29.8	7.4	50.0
**7 to 9**	***stx1***	9.0	-5.3	0.105	0.58	7.4	12.8	1.6	78.2
**Months**	***stx2***	7.4	-18.6	0.000**	0.24	5.9	24.5	1.6	68.1
**August**	***stx1/2***	3.7	3.2	0.033*	7.00	3.7	0.5	0.0	95.7
**Sample IV**	**STEC**	20.2	0.0	1.000	1.00	16.0	16.0	4.3	63.8
**10 to 12**	***stx1***	1.6	-7.4	0.002**	0.18	1.6	9.0	0.0	89.4
**Months**	***stx2***	14.9	7.4	0.027*	2.08	14.4	6.9	0.5	78.2
**December**	***stx1/2***	3.7	0.0	1.000	1.00	3.7	3.7	0.0	92.6

*a* Prevalence of STEC determined by screening of fecal sample using Polymerase Chain Reaction (PCR)

*b* Percentage of cattle that gained (-)/(+) or lost (+)/(-) colonization by STEC; remain colonized (+)/(+) or uncolonized (-)/(-)

*c* Statistical significance of the change in STEC prevalence between calf ages given by McNemar's test;

* Denotes statistical significance at α = 0.05

** denotes statistical significance at α = 0.01

*d* Likelihood of becoming colonized (Odds Ratio) with STEC among sampling times

### Metagenomic Analysis Revealed a More Diverse Microflora in Older Cattle

**Fig 6:**
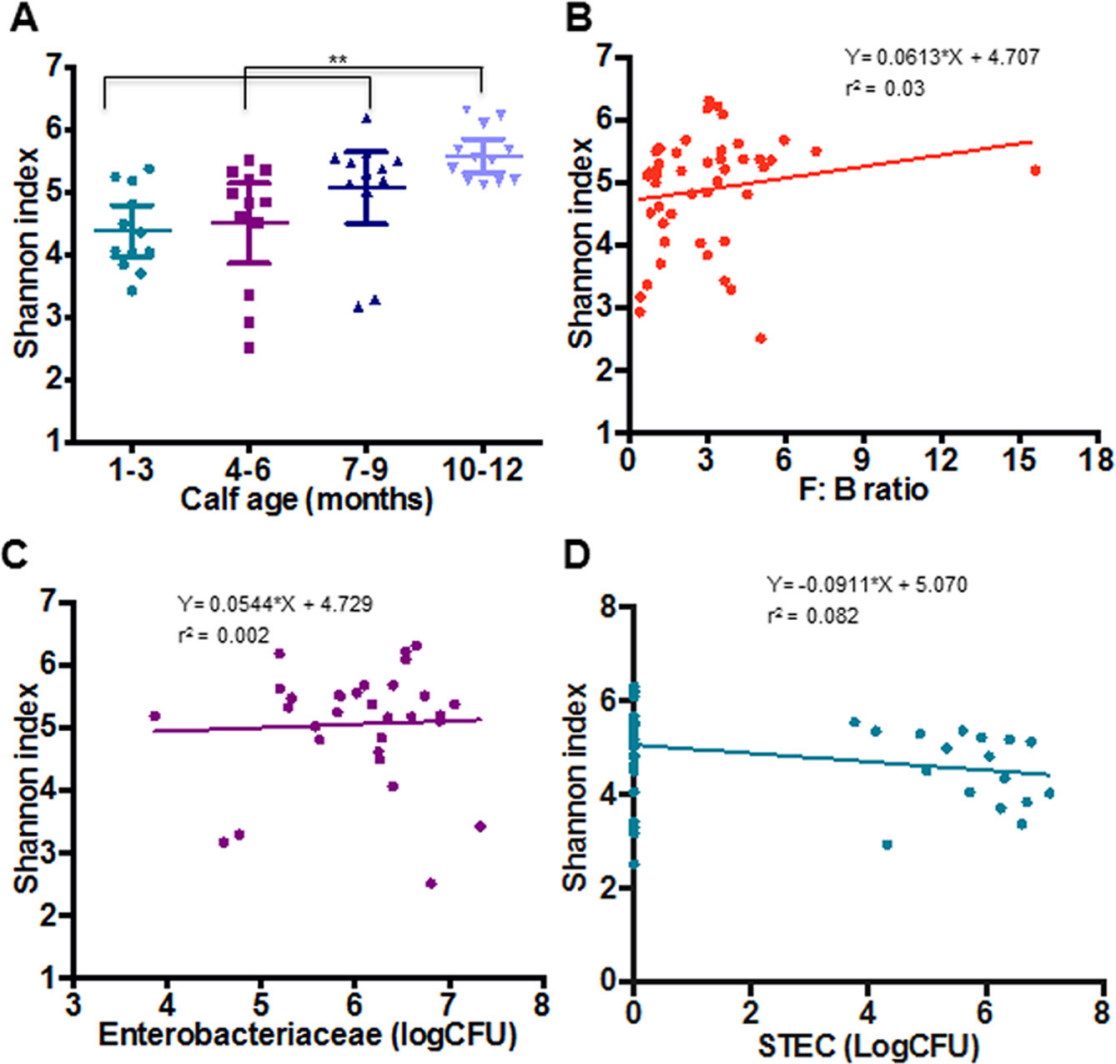
Metagenomic analysis of fecal samples collected at four different ages from beef calves. (A) The microbial diversity of fecal samples expressed by the Shannon index was determined and compared in four age groups by linear regression. (B) Linear regression between Shannon index and the Firmicutes: Bacteroides (F:B) ratio, which indicates the abundance of healthy microflora. (C) Linear regression between Shannon index and the abundance of *Enterobacteriaceae* (the beneficial microflora). (D) Linear regression between Shannon index and the abundance of STEC (Log CFU) (colony forming units).

**Fig 7:**
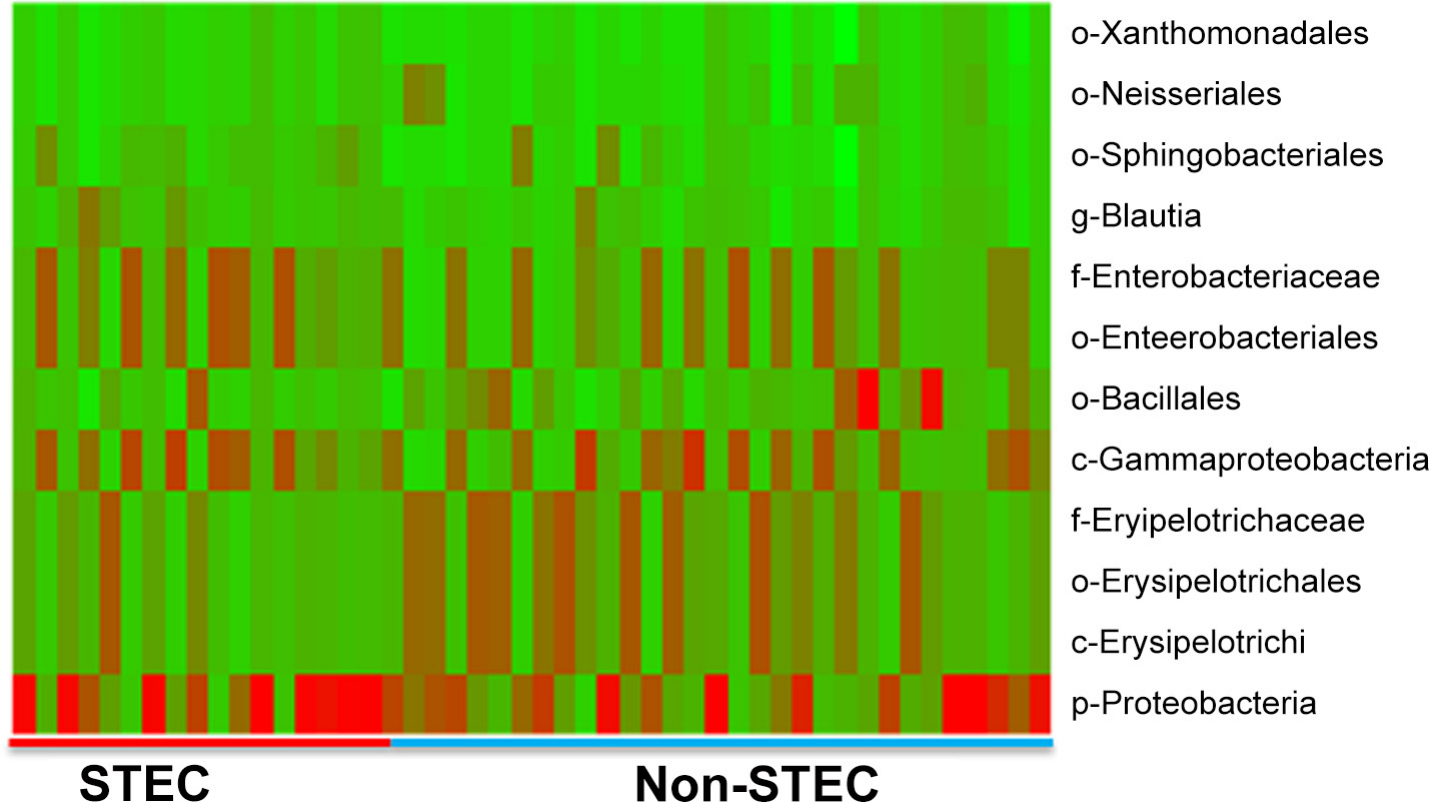
Heat map showing difference in microflora in STEC positive and STEC negative samples. The G-plot package of R-program was sued to generate a heat map from the data of abundance of OTUs in fecal samples; (p = phylum, c = class, o = order, f = family and g = genus).

In order to determine the role of fecal microflora in STEC dynamics, DNA collected from the fecal samples of calves was analyzed using 454 pyrosequencing. Shannon index has been used to measure microbial diversity [17] and the relative abundance of Firmicutes (F) and Bacteroidetes (B) have been used to measure overall animal health and production traits. It has been shown that the higher F:B ration is positively associated with milk production and animal health [18, 19]. In this study, we identified that the Shannon diversity index was higher in fecal samples from older animals (*P* = 0.0074) (Fig 6A) and increased linearly with the F:B ratio (Fig 6B). Samples collected from calves not colonized by STEC had a higher Shannon index (*P* = 0.073) (Fig 6C), which decreased as the concentration of STEC increased (Fig 6D). Calves shedding *stx1* or *stx1/stx2* genotypes had a higher percentage of the phylum Bacteroidetes (*P* = 0.011) and class Bacteroidia (*P* = 0.0704). Some of the bacterial taxonomic units such as Proteobacteria, Gammaproteobacteria, Sphingobacteriales, Enterobacteriales, were more prevalent in STEC positive samples (*P*<0.05), while the STEC negative samples had a higher proportion containing Erysipelotrichi, Xanthomonadales, Bacillales, Neisseriales (*P*<0.05) (Fig 7) (Table 2). It was also observed differences in the abundance of various Operational Taxonomic Units (OTUs) between male and female calves (S1 Table and S3 Fig), indicating that microflora might be related to animal sex. Only few of the OTUs were different among breed groups (S4 Fig), with Breed 1 having higher Firmicutes (*P* = 0.0786), Tenericutes (*P* = 0.0343) and Lentisphaerae (*P* = 0.0021) (S2 Table and S5 Fig). The OTUs that were identified at 1–3 months age were significantly different (*P*<0.05) from the OTUs identified at 10–12 months age (S3 Table). For example, older calves had higher Spirochaetes (*P* = 0.0039) while the younger calves had higher Desulfovibrionales (*P* = 0.00132) and Proteobacteria (*P* = 0.0003).

**Table 2:** Metagenomic analysis of fecal samples from both STEC positive and STEC negative calves^a^.

Non-STEC		STEC	
Aboundant OTUs	*P*- value^*b*^	Aboundant OTUs	*P*- value^*b*^
c- Erysipelotrichi	0.0471	p- Proteobacteria	0.0452
o- Xanthomonadales	0.0232	c- Gammaproteobacteria	0.0214
o- Neisseriales	0.0101	o- Sphingobacteriales	0.0188
o- Bacillales	0.0143	o- Enterobacteriales	0.022
o- Erysipelotrichales	0.0469	f- Enterobacteriaceae	0.0332
f- Erysipelotrichaceae	0.0469	g- Blautia	0.0102

*a* Mean proportions of the bacterial taxa (OTUs) within the fecal samples of beef calves

*b* Abundance of OTUs were statistically analyzed using a generalized linear mixed model in SAS, statistical significance calculated at α = 0.05

OTUs = Operational Taxonomical Units; p = Phylum; c = Class; o = Order; f = Family; g = Genus

In the fecal samples analyzed in this study the F: B ratio was higher in older calves (S1 Fig) and lower in STEC positive animals, with respect to both the prevalence (*P* = 0.043), and the concentration (log CFU) of STEC (*P* = 0.0468). STEC negative samples had a higher F:B ratio (S2 Fig) which increased linearly, but not significantly with the concentration of *Enterobacteriaceae* (*P* = 0.83). The Shannon index increased linearly with F: B ratio (*P* = 0.246) (Fig 6B) and was higher, but not statistically significant in STEC negative samples (*P* = 0.073). No significant differences were found in the Shannon index among different breed groups or sex categories of the calves.

## Discussion

By following a cohort of beef calves born in the same calving period from a single farm, we were able to gain novel insights into the dynamics of STEC colonization. Utilization of metagenomic analyses also allowed for the first time an investigation of the role of gut microflora on the dynamics of STEC colonization.

Though in the previous study [10] heifers were identified to have a lower prevalence of STEC compared to older cattle, further analysis during the first year of life revealed that the highest rates of STEC shedding were observed during the first 6 months. In agreement with the previous study, by the end of the first year of life only approximately 20% of cattle were colonized by STEC. Interestingly, the major STEC genotype isolated from 12 months old calves was *stx2*. Two *stx* genes encode Shiga toxins which are *stx1AB* and *stx2AB*, and it has been reported that Stx2 is associated with more severe human infections [20, 21]. The predominance of *stx2* genotype in older calves is noteworthy, although the overall STEC prevalence declined when animals aged, because Stx2 is 1000 times more toxic than Stx1 as shown previously in animal models [22, 23]. In addition, recent Shiga toxin producing *E*. *coli* O157:H7 outbreak strains have been found to carry two copies of the *stx2AB* gene cluster instead of one of the *stx1AB* and one of the *stx2AB* clusters each, making them more virulent than the isolates carrying only the *stx1AB* gene cluster, due to lack of competition for GB3 binding [20, 21]. Taken together, although the prevalence of STEC was lower in older animals, the calves were carrying more pathogenic STEC, suggesting further STEC genotyping is necessary for proper risk assessment.

Genome wide analysis has shown that STEC O157 can be grouped into three main phylogenetic lineages, called lineages I, II and I/II. Lineage I isolates originate from human clinical and bovine sources, while a majority of lineage II isolates have bovine origins. Lineage I/II includes strains isolated from human infections and hyper-virulent strains that include a multistate spinach outbreak [8, 24, 25]. These three STEC O157 lineages carry a genetic signature in which each lineage encodes unique *stx* genes. *stx1* and *stx2a* are more frequently identified in lineage I; whereas, *stx1* and *stx2c* are detected in lineage II and *stx2a* and *stx2c* are predominantly identified from lineage II [24]. In this study, we have evaluated the dynamic prevalence of variant STEC subtypes of *stx1*, *stx2*, and/or *stx1/2* during animal aging. Further analysis to differentiate *stx2* subtypes could provide clues to assign STEC isolates into the three lineages that may help predict potential burden of the isolates in cattle.

In this study we investigated other physiological factors such as breed, sex and average weight gain for their role in STEC shedding, but were unable to find any significant relationships between these factors and STEC colonization. However, the metagenomic analyses indicated that older calves have a more diverse microflora that was associated with a reduced prevalence of STEC as the calves matured. The correlation between increased diversity of microflora and STEC prevalence was confirmed in a second year of sample collection using a separate group of animals. The results from the second year of sampling also indicated that there was no significant association between breed, sex or weight gain of animal and STEC prevalence.

The low concentration of normal *Enterobacteriaceae* microflora coupled with higher concentrations of STEC in the early stages of life may be explained by the lower concentration and diversity of commensal microflora in the gut of the young calves, which increases with age [26, 27]. The microflora could have also been affected by the diet of the animals [28], which changes as the animal matures, weans, and begins grazing in the pasture. Thus, the animal diet might be one mechanism by which the microflora and the colonization of STEC are associated with the age of the animals. In the current study, it was also observed that the Shannon index increased as the calves mature and was associated with a lower STEC prevalence in older claves. No significant differences in the Shannon index were observed between breed groups or between male and female calves. Previously, a higher F: B ratio was associated with a more diversified microflora, which lead to better health and production traits of cows [18, 29]. Since the results of this study indicated that the Shannon index was linearly correlated with the F:B ratio, our hypothesis that older calves have more diverse microflora which reduces STEC colonization was supported by the metagenomics analyses.

The prevalence of STEC has been suggested to increase markedly after weaning (16.6% to 38.3%) and remain elevated during the pre-conditioning period in high moisture fed calves due to stresses caused by diet change [30]. Similar results were also reported in dairy calves where the shedding of STEC and *E*. *coli* O157 increased soon after weaning [31]. However, in this study, evaluated with beef calves that were weaned around 6–8 months of age onto a pasture with free access to water, no significant increase in the STEC shedding was identified after weaning in both years. This might be the result of the access to grazing on loose pasture that reduces animal density and prevents transmission of STEC from other animals, which could have led to a reduction in the shedding of STEC by weaned calves. Another possible reason for the increased prevalence during the first sampling period was the waning of maternal antibodies and subsequent colonization, followed by the development of the calf’s own antibodies to STEC. In dairy calves, it has been reported that colostrum antibodies against *stx1* and *stx2* reach peak titers within first 24 hours of life and *stx1* antibody can be detected up to four weeks [32]. However, since antibodies were not investigated in this study, the effect of maternal or acquired immunity was not able to be determined and could be a focus of future research.

## Supporting Information

S1 Fig Metagenomic analysis (F:B ratio) showing difference in microflora of fecal samples collected from young and old beef calves. (PDF)

S2 Fig Metagenomic analysis (F:B ratio vs Enterobacteriaceae) showing association between normal microflora and F: B ratio in beef calves. (PDF)

S3 Fig Heat map of abundance of microflora in fecal samples collected at different ages from calves. (TIF)

S4 Fig Heat map of abundance of microflora in fecal samples collected from male and female calves. (TIF)

S5 Fig Heat map of abundance of microflora in fecal samples collected from six breed groups of beef calves. (TIF)

S1 Table Metagenomic analysis of fecal samples from male and female beef calves^*a*^. (PDF)

S2 Table Metagenomic analysis of fecal samples from calves in different breed groups^*a*^. (PDF)

S3 Table Metagenomic analysis of fecal samples in different age groups of the calves^*a*^. (PDF)
