# Supplementary figures and images for: Colonization of Beef Cattle by Shiga Toxin-Producing Escherichia coli during the First Year of Life: A Cohort Study
Source: PLoS One. 2016 Feb 5;11(2):e0148518. doi: 10.1371/journal.pone.0148518 (PMC4743843; doi:10.1371/journal.pone.0148518)

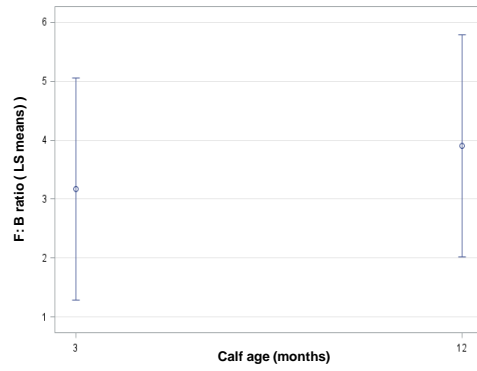

Supplement: S1 Fig — (PDF) [file pone.0148518.s001.pdf]

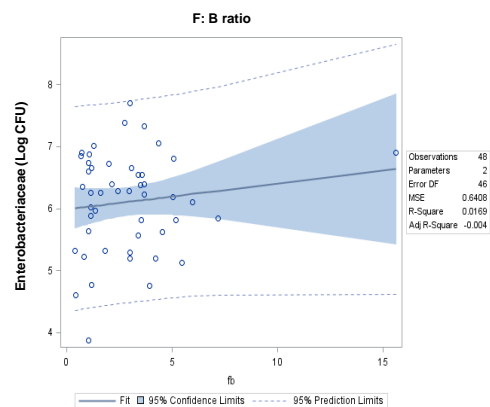

Supplement: S2 Fig — (PDF) [file pone.0148518.s002.pdf]

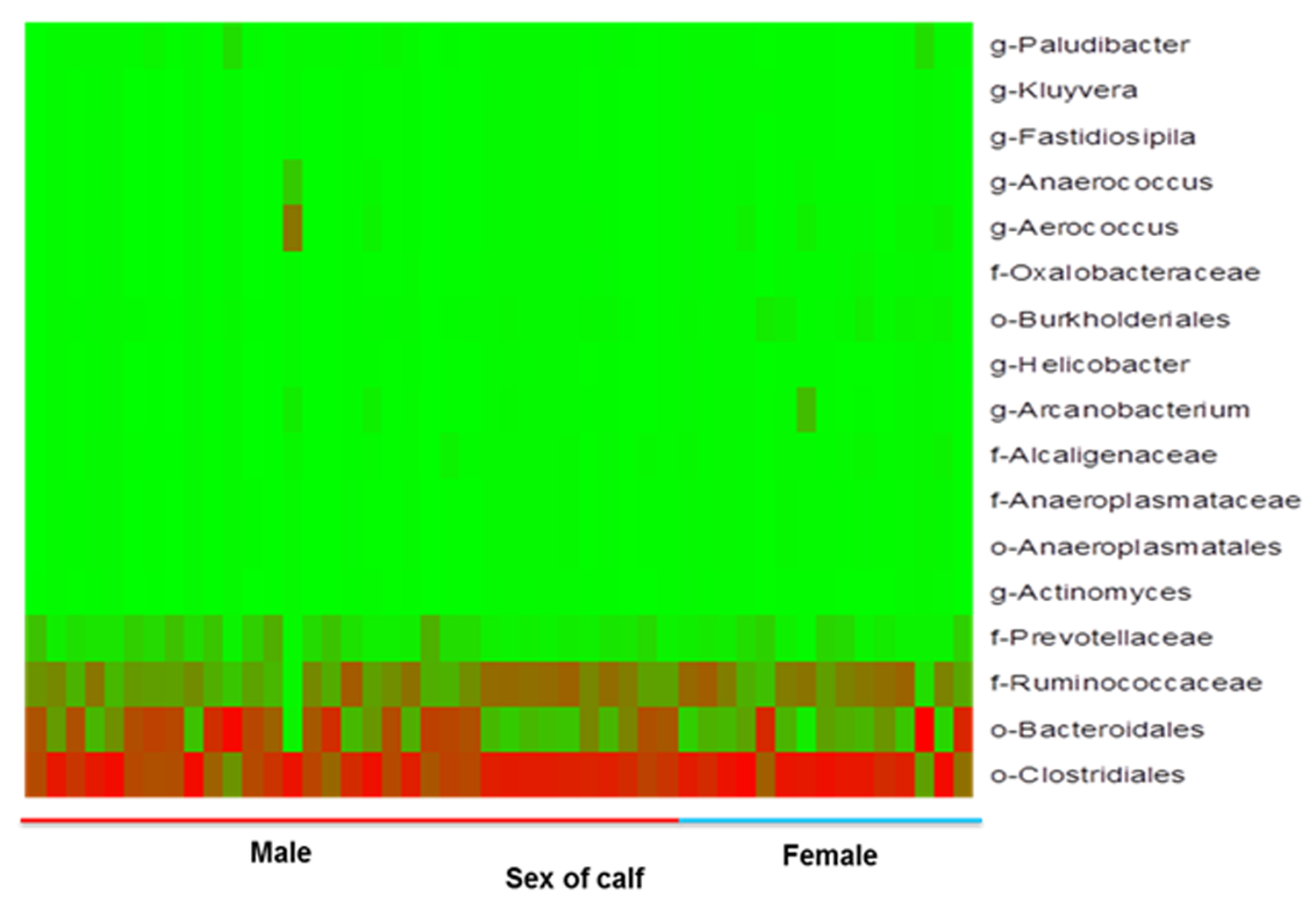

Supplement: S3 Fig — (TIF) [file pone.0148518.s003.tif]

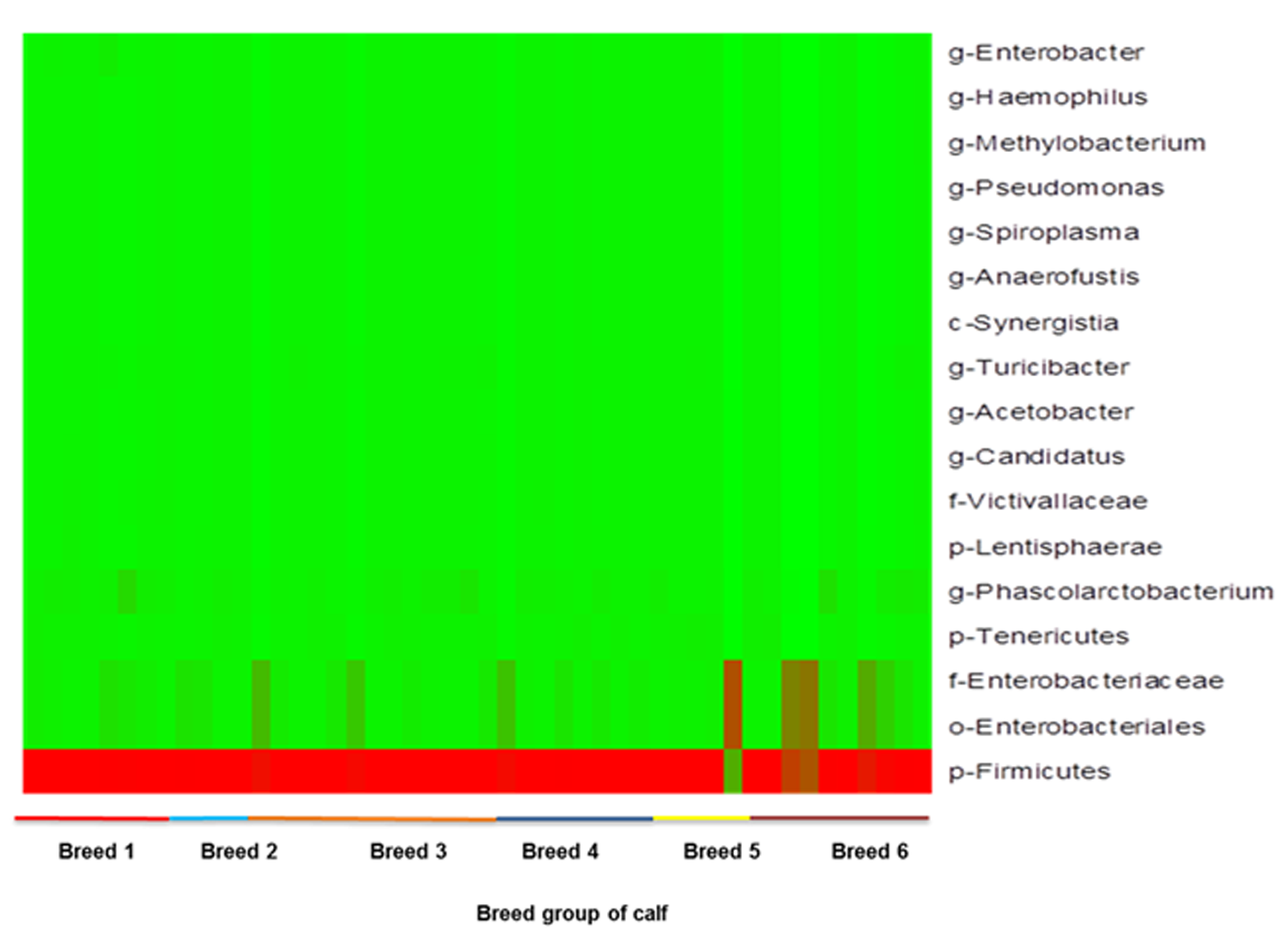

Supplement: S4 Fig — (TIF) [file pone.0148518.s004.tif]

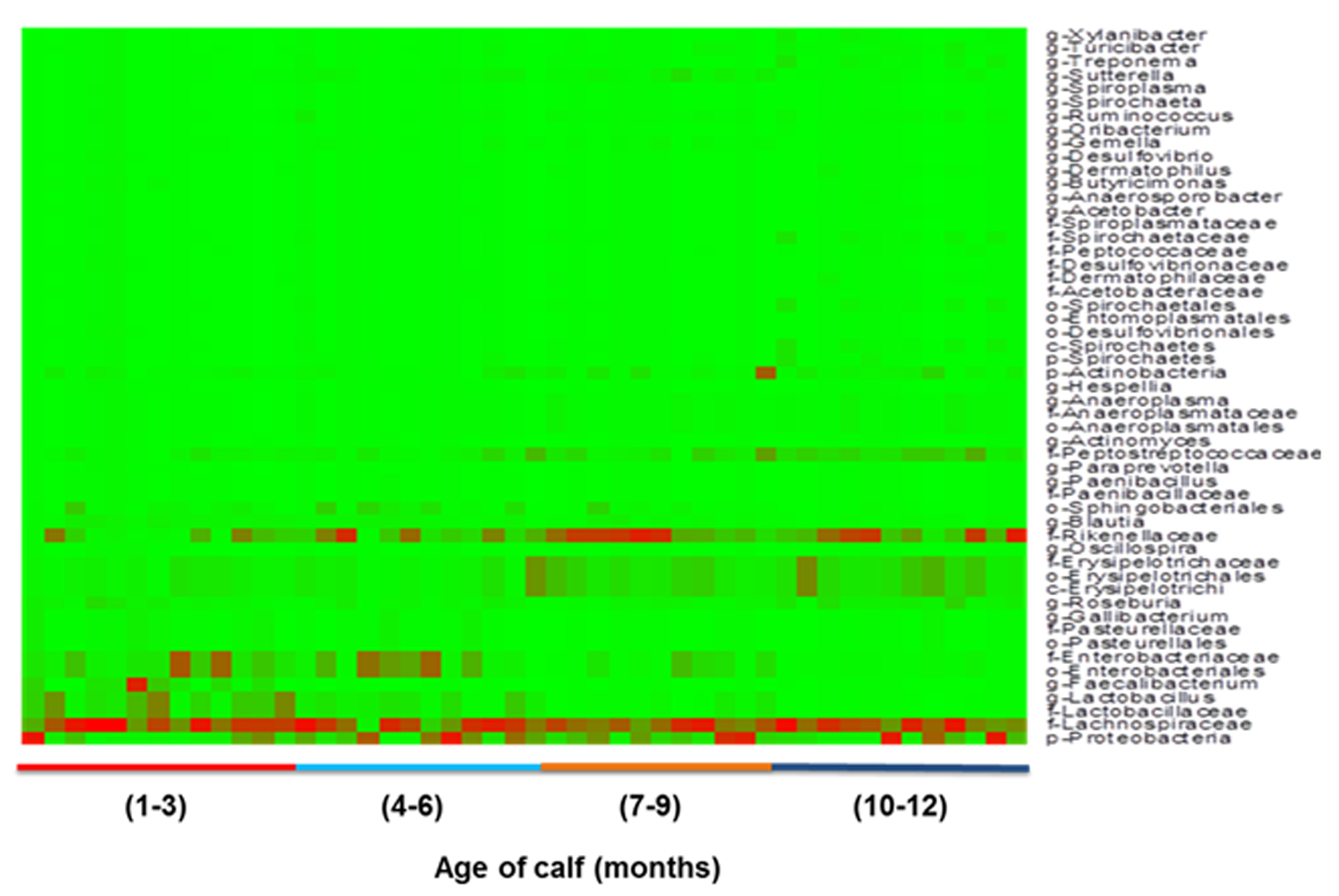

Supplement: S5 Fig — (TIF) [file pone.0148518.s005.tif]
