# Supplementary material for: Colonization of Beef Cattle by Shiga Toxin-Producing Escherichia coli during the First Year of Life: A Cohort Study
Source: PLoS One. 2016 Feb 5;11(2):e0148518. doi: 10.1371/journal.pone.0148518 (PMC4743843; doi:10.1371/journal.pone.0148518)
Supplement: S1 Table — (PDF) [file pone.0148518.s006.pdf]

S1 Table: Metagenomic analysis of fecal samples from male and female beef calves[a]

Males		Females	
**Aboundant OTUs**	***P*- value**[b]	**Aboundant OTUs**	***P*- value**[b]
o- Bacteroidales	0.0069	o- Burkholderiales	0.011
f- Prevotellaceae	0.0015	o- Anaeroplasmatales	0.0494
g- Fastidiospila	0.022	o- Clostridiales	0.0347

a Mean proportions of the bacterial taxa (OTUs) within the fecal samples of male and female calves
b Abundance of OTUs were statistically analyzed using a generalized linear mixed model in SAS, statistical significance calculated at $\alpha = 0.05$
OTUs= Operational Taxonomical Units; p= Phylum; c= Class; o= Order; f= Family; g= Genus
