# Supplementary material for: Colonization of Beef Cattle by Shiga Toxin-Producing Escherichia coli during the First Year of Life: A Cohort Study
Source: PLoS One. 2016 Feb 5;11(2):e0148518. doi: 10.1371/journal.pone.0148518 (PMC4743843; doi:10.1371/journal.pone.0148518)
Supplement: S2 Table — (PDF) [file pone.0148518.s007.pdf]

S2 Table: Metagenomic analysis of fecal samples from calves in different breed groups[a]

Breed Group	Aboundant OTUs	*P*- value[b]
**Breed 1**	p- Firmicutes	0.0786
	p- Tenericutes	0.0343
	p- Lentisphaerae	0.0021
	g- Phascolarctobacterium	0.0397
**Breed 2**	c- Synergistia	0.0581
	o- Enterobacteriales	0.0392
	g- Turibacter	0.0180
	g- Spiroplasma	0.0422
**Breed 3**	g- Methylobacterium	0.0415
**Breed 4**	f- Enterobacteriaceae	0.0307
	g- Methylobacterium	0.0415
	g- Anaerofustis	0.0415
	g- Phascolarctobacterium	0.0397
**Breed 5**	f- Victivallaceae	0.0832
	g- Candidatus	0.027
	g- Phascolarctobacterium	0.0397
**Breed 6**	f- Victivallaceae	0.0832

a Mean proportions of the bacterial taxa (OTUs) within the fecal samples of calves from different breed groups
b Abundance of OTUs were statistically analyzed using a generalized linear mixed model in SAS, statistical significance calculated at $\alpha = 0.05$
OTUs= Operational Taxonomical Units; p= Phylum; c= Class; o= Order; f= Family; g= Genus
