# Supplementary material for: Colonization of Beef Cattle by Shiga Toxin-Producing Escherichia coli during the First Year of Life: A Cohort Study
Source: PLoS One. 2016 Feb 5;11(2):e0148518. doi: 10.1371/journal.pone.0148518 (PMC4743843; doi:10.1371/journal.pone.0148518)
Supplement: S3 Table — (PDF) [file pone.0148518.s008.pdf]

S3 Table: Metagenomic analysis of fecal samples in different age groups of the calves[a]

1-3 months		4-6 months		7-9 months		10-12 months	
**Aboundant OTUs**	***P*- value[b]**	**Aboundant OTUs**	***P*- value[b]**	**Aboundant OTUs**	***P*- value[b]**	**Aboundant OTUs**	***P*- value[b]**
o- Desulfovibrionales	0.0132	p- Proteobacteria	0.0003	f- Lachnospiraceae	0.0064	p- Spirocahetes	0.0039
o- Entomoplasmatales	0.0072	o- Sphinobacteriales	0.028	g- Dermatophilus	0.005	p- Actinobacteria	0.0799
o- Pasteurellales	0.0155	o- Enterobacteriales	0.0208	g- Sutterella	0.0062	c- Spirochaetes	0.0039
o- Sphinobacteriales	0.028	f- Paenibacillaceae	0.0057	g- Gemella	0.0081	c- Erysipelotrichi	0.003
o- Enterobacteriales	0.0208	f- Pasteurellaceae	0.0064	g- Desulfurvibrio	0.0148	o- Spirochaetales	0.0038
f- Spiroplasmataceae	0.0089	f- Lachnospiraceae	0.0064			o- Anaeroplasmatales	0.0028
f- Desulfovibrionaceae	0.0134	g- Gemella	0.0081			o- Erysipelotrichales	0.003
f- Paenibacillaceae	0.0057	g- Paenibacillus	0.0065			f- Peptococcaceae	0.013

a Mean proportions of the bacterial taxa (OTUs) within the fecal samples of different age groups of beef calves

b Abundance of OTUs were statistically analyzed using a generalized linear mixed model in SAS, statistical significance calculated at $\alpha = 0.05$

OTUs= Operational Taxonomical Units; p= Phylum; c= Class; o= Order; f= Family; g= Genus
